# Systematical analysis reveals a strong cancer relevance of CREB1-regulated genes

**DOI:** 10.1186/s12935-021-02224-z

**Published:** 2021-10-12

**Authors:** Tianyu Zheng, Jinrong Huang, Xi Xiang, Siyuan Li, Jiaying Yu, Kunli Qu, Zhe Xu, Peng Han, Zhanying Dong, Yang Liu, Fengping Xu, Huanming Yang, Marja Jäättelä, Yonglun Luo, Bin Liu

**Affiliations:** 1 College of Life Sciences, University of Chinese Academy of Sciences, Beijing, 100049 China; 2 Lars Bolund Institute of Regenerative Medicine, Qingdao-Europe Advanced Institute for Life Sciences, BGI-Qingdao, BGI-Shenzhen, Qingdao, 266555 China; 3 Department of Neuroscience, Karolinska Institutet, 171 77 Stockholm, Sweden; 4 BGI-Shenzhen, Shenzhen, China 518083; 5 Department of Biomedicine, Aarhus University, 8000 Aarhus, Denmark; 6 Department of Biology, University of Copenhagen, Copenhagen, Denmark; 7 Steno Diabetes Center Aarhus, Aarhus University Hospital, Aarhus, Denmark; 8 Cell Death and Metabolism, Center for Autophagy, Recycling and Disease, Danish Cancer Society Research Center, 2100 Copenhagen, Denmark

**Keywords:** CREB1, Cancer, Transcription factor, RRHO, Kaplan–Meier analysis

## Abstract

The transcription factor cyclic-AMP response element-binding protein 1 (CREB1) responds to cAMP level and controls the expression of target genes, which regulates nutrition partitioning. The promoters of CREB1-targeted genes responsive to cAMP have been extensively investigated and characterized with the presence of both cAMP response element and TATA box. Compelling evidence demonstrates that CREB1 also plays an essential role in promoting tumor development. However, only very few genes required for cell survival, proliferation and migration are known to be constitutively regulated by CREB1 in tumors. Their promoters mostly do not harbor any cAMP response element. Thus, it is very likely that CREB1 regulates the expressions of distinct sets of target genes in normal tissues and tumors. The whole gene network constitutively regulated by CREB1 in tumors has remained unrevealed. Here, we employ a systematical and integrative approach to decipher this gene network in the context of both tissue cultured cancer cells and patient samples. We combine transcriptomic, Rank-Rank Hypergeometric Overlap, and Chipseq analysis, to define and characterize CREB1-regulated genes in a multidimensional fashion. A strong cancer relevance of those top-ranked targets, which meet the most stringent criteria, is eventually verified by overall survival analysis of cancer patients. These findings strongly suggest the importance of genes constitutively regulated by CREB1 for their implicative involvement in promoting tumorigenesis.

**Supplementary Information:**

The online version contains supplementary material available at 10.1186/s12935-021-02224-z.

## Background

CREB1(cAMP response element-binding protein) belongs to a family of transcription factors whose activities are induced by increased intracellular cAMP [1, 2]. The protein consists of a kinase-inducible domain, two glutamine-rich domains, and a bZIP domain [1]. Upon PKA-mediated reversible phosphorylation at serine 133 [3], CREB1 binds to DNA via a bZIP domain that recognizes cAMP response element (CRE), TGACGTCA, and half CRE, TGACG/CGTCA [4, 5]. The cAMP responsiveness of CREB1 target genes, whose promoters contain CRE and half CRE, is also dependent on the presence of TATA box in their promoters [6]. Although more than 4000 CREB1 affinity bonded promoters have been identified by Chip-seq, only 339 genes’ promoters contain both CRE and TATA box, and less than 100 genes are validated as CREB1 target genes in a cAMP-responsive manner [7]. This suggests that many other putative CREB1 target genes might be regulated in a cAMP-independent or even CRE-independent manner. Moreover, given that many transcription factors are downstream targets of CREB1 [7], the gene network regulated by CREB1 tends to be even more complex.

In accordance with this complexity, CREB1 has pleiotropic functions and is implicated in many physiological and pathological processes, e.g., muscle hypertrophy [8], metabolic adaptions to exercise [8], mitochondrial biogenesis [9], beta cell fitness [10]. Importantly, CREB1 also plays a role in cancers, and its expression level is associated with the overall survival and therapy response of tumor patients. Increased expression of *CREB1* has been reported in acute lymphoblastic leukemia [11], acute myeloid leukemia [12], melanoma [13], hepatocellular [14], renal cell [15], ovarian [16], prostate [17], lung [18], and breast carcinoma [19], brain tumors [20], etc. compared to their counterpart healthy tissues [2]. CREB1-regulated target genes promote cell proliferation, migration, survival, and chemotherapy resistance in cancer cells. Consistently, the most frequent consequences of suppressing CREB1 by either knockdown or inhibitors are cell death, metastatic defect, failure to form colony and filopodia, and inhibition of tumor growth [15, 20–23]. Contrary to the widely acknowledged essential role of CREB1 in cancers, a comprehensive and in-depth investigation of the gene network mediated by CREB1 is less explored. Only a limited number of CREB1-targeted genes, including *CCNA1, CCND1, BCL2, MMP2, MMP9*, *GSK3A* [12, 20, 21, 24–26], were shown to contribute to tumor development. Many of them are tumor type-specific. For example, *CCNA1* is a target of CREB1 and is upregulated in acute myeloid leukemia while BCL2 is not [12]. However, BCL2 is a validated CREB1 target and promotes cell survival in glioma cells [20]. Such kind of discrepancies is important for understanding distinct functions of CREB1 in different types of cancer and providing guidance for tailored anti-tumor strategies. In addition, as the classic function of CREB1 is to regulate nutrient partitioning in metabolic tissues [8], most previous studies about CREB1 targets mainly focus on those genes that are cAMP-responsive [6, 7]. Whereas in cancer cells, there might be more genes constitutively regulated by CREB1 regardless of cAMP activation. *CCNA1* is a typical example whose promoter has no CRE. But its expression is significantly increased by overexpression of CREB1 in leukemia cells [12]. Thus, the gene network mediated by CREB1 in tumors could be rather distinct from normal tissues.

To investigate this complex gene network and identify essential CREB1 target genes with strong cancer relevance in a systematic manner, we designed and performed the present study. Traditional strategies to uncover the gene network mediated by a transcription factor mainly rely on transcriptomic and Chipseq analyses. The cancer relevance of each target gene needs to be accessed by examining various cancerous hallmarks after modulating the expression level of the target gene in preclinical models. Due to a large number of target genes and a variety of cancer types, this is a long-lasting process, ranging from 3 to 10 years. We develop a novel strategy with a more straightforward and shortened workflow. Firstly, transcriptomic analysis of HeLa (cervical cancer) cells with CREB1 deficiency or rescued CREB1 expression was carried out to identify the CREB1-mediated gene network. The cancer relevance of these genes was determined by a Rank-Rank Hypergeometric Overlap (RRHO) analysis with patients’ transcriptomic data from TCGA. We further categorized CREB1-regulated genes into different groups according to Chiqseq, RNAseq, and RRHO analysis and confirmed the significances of several novel CREB1 target genes, whose expressions matter for the overall survival of cancer patients. Consistent with our initial hypothesis, most of the top-ranked CREB1 target genes have no CRE. This strongly underscores the novelty, necessity, and importance of our present study.

## Results

### Generation of CREB1 knockout (KO) cells and the corresponding CREB1 rescue cells

**Fig. 1:**
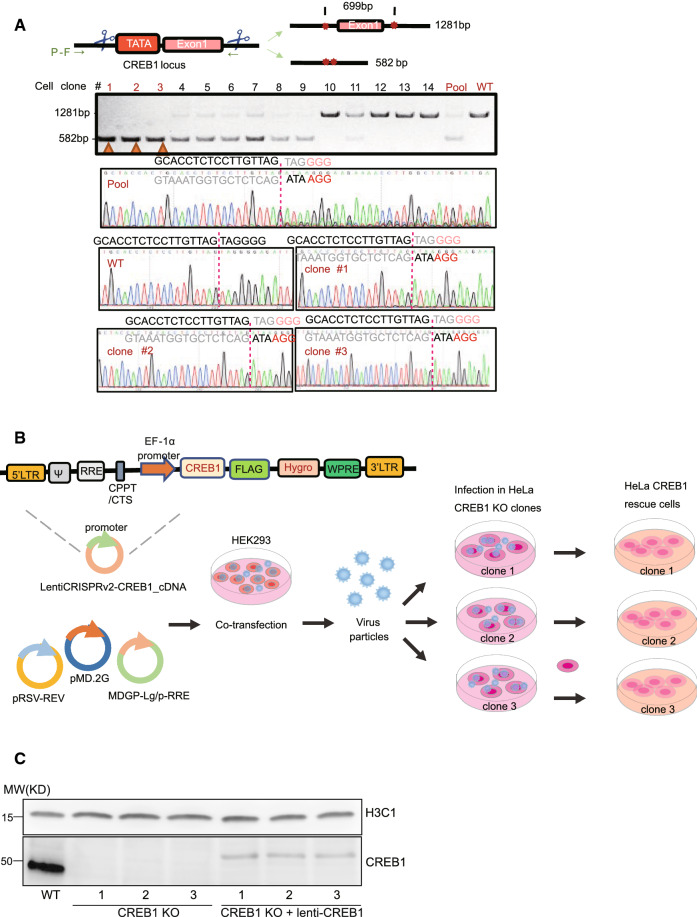
The generation of CREB1 knockout (KO) cells and rescue cells. **A** The generation of CREB1 knockout cells by CRISPR/Cas9 technology. A pair of gRNAs were designed to target the upstream and downstream sequences of CREB1-exon 1. After puromycin selection, CREB1 knockout clones carrying homozygous deletion of exon 1 were screened out by PCR. Clone 1–3, pool (all clones mixture), and WT cells were verified by Sanger sequencing. P–F and P–R indicate the locations of the targeted sequence by gRNA. **B** The generation of CREB1 rescued cells. Lenti CREB1 Hygro plasmid was constructed by cloning CREB1 cDNA and EF1α-driven HygroR into LentiCRISPRv2 plasmid. Lenti CREB1 Hygro was co-transfected with three packaging plasmids into HEK293T cells. Virus particles were collected and used to infect 3 different CREB1 knockout clones. Finally, CREB1 rescued cells were obtained after hygromycin selection. C. Western blot of indicated proteins in wild type (WT), CREB1 KO, and CREB1 rescue cells

To identify a transcription factor-regulated gene network, comparing expression patterns between cells with and without the transcription factor is an effective and routine methodology. This becomes particularly handy because of the CRISPR/Cas9 technology [27]. However, due to the essential function of CREB1 in normal and some cancer cells, CREB1 knockout via CRISPR/Cas9 has not been quite successfully established [2]. To circumvent this dilemma, we chose an experimentally robust cervical cancer cell line (HeLa), which has a comparable expression of CREB1 to many CREB1 sensitive cancer cell lines, to generate the CREB1 KO cells. We adopted a more efficient and predictive CRISPR knockout strategy [28], and two sgRNAs were designed to target *CREB1* intron 1 and the proximal promoter (including the TATA box) (Fig. 1A). After screening 14 clones, we obtained three parallel HeLa CREB1 KO clones with deletion of all alleles (Fig. 1A). To rescue CREB1 expression in the KO cells, we constructed a lentiviral expression plasmid of CREB1 (Fig. 1B). Following a standard infection method, we obtained corresponding CREB1 rescue cells for all three KO clones (Fig. 1B). Western blot further confirmed the absence and presence of CREB1 protein in the KO and rescue cell lines respectively (Fig. 1C).

### Gene expression analysis of wild type (WT) cells, CREB1 KO cells, and the corresponding CREB1 rescue cells

To identify the CREB1-mediated gene network in HeLa cells, we performed RNA sequencing (RNAseq) and transcriptomic analysis of the above-obtained cells and control WT cells. Hierarchical clustering heatmap showed high similarities among the replicates of the same clone (Additional file 1: Figure S1A). Moreover, principal components analysis clearly separated WT cells from KO cells, and rescue cells from KO cells (Additional file 1: Figure S1B), indicating that the transcriptomic alteration is genotype-dependent.

**Fig. 2:**
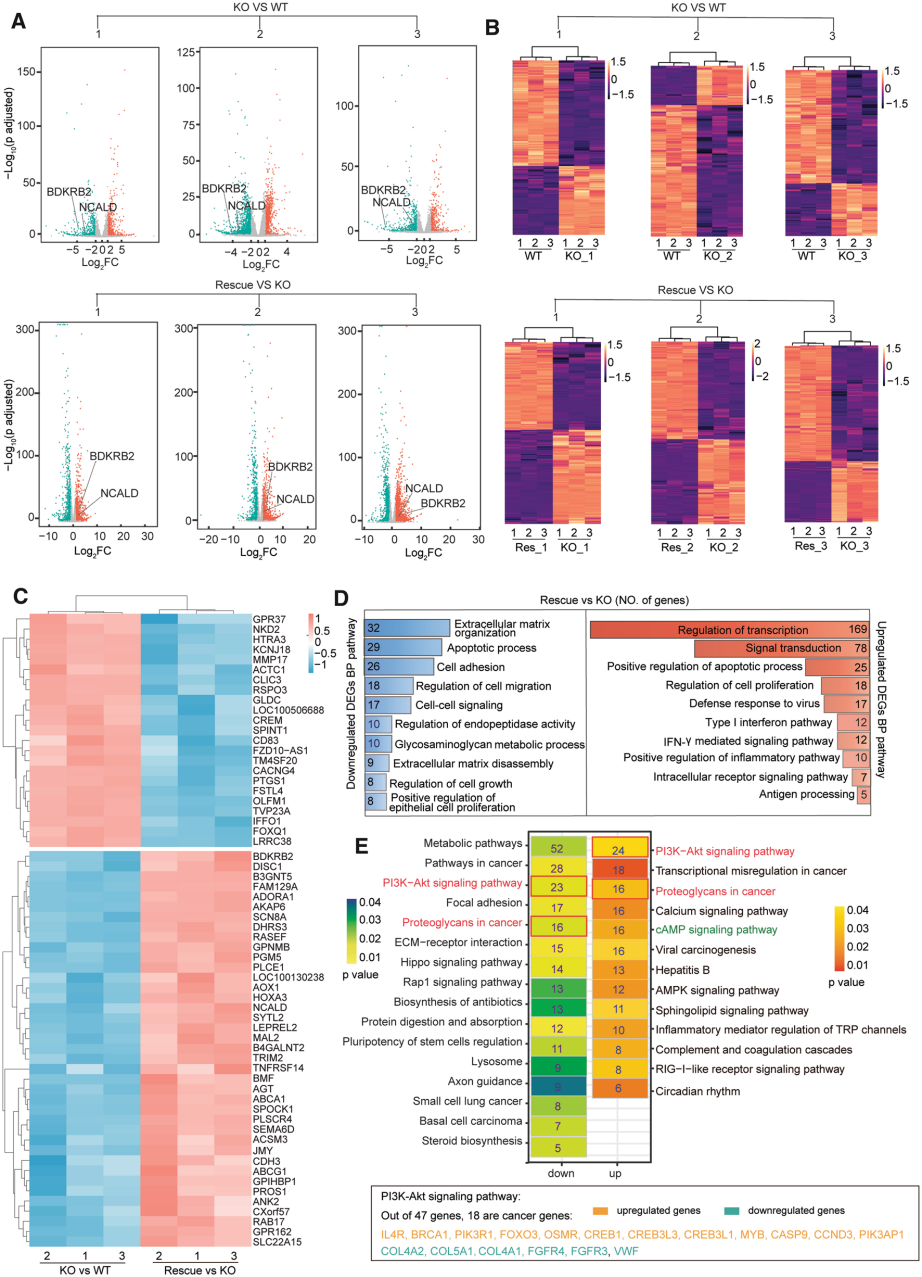
RNA-seq analysis of CREB1 KO cells, CREB1 rescue cells, and WT cells. **A** Volcano plots of CREB1 knockout (KO) clone 1–3 vs WT and CREB1 rescue clone 1–3 vs CREB1 KO clone 1–3. Differential gene expression profiles are displayed according to log2 Fold Change (log2 FC) and p adjusted (Additional file 3: Table S2). The threshold of differentially expressed genes (DEGs) was set to p-adjusted ≤ 0.05 and |log_2_ FC| ≥ 1. **B** Heatmaps of DEGs in indicated pairwise comparisons. Res is short for rescue. **C** Heatmaps of DEGs with opposite expression profiles between CREB1 KO vs WT and Rescue vs KO. Twenty three genes were upregulated in all CREB1 KO cell lines (compared to WT) and downregulated in all CREB1 rescue cell lines (compared to KO), while 39 genes were downregulated in all CREB1 KO cell lines and upregulated in all CREB1 rescue cell lines. **D** Gene Ontology (GO) analysis of DEGs in CREB1 commonly regulated gene list (genes showing similar differential expression patterns in at least two pairs of rescue clones and corresponding KO clones) (Additional file 3: Table S2). It contains both upregulated and downregulated genes. The X-axis shows the NO. of genes in the enriched GO terms. The Y-axis shows the top 10 GO terms of biological process. All GO terms were selected by p value ≤ 0.01. E. KEGG (Kyoto Encyclopedia of Genes and Genomes) pathway analysis of DEGs in CREB1 commonly regulated gene list. The NO. in each rectangle shows NO. of genes enriched in the corresponding pathway. The scale of p value is displayed by the gradient bar. The pathways highlighted by red boxes are enriched in both down and upregulated DEGs lists. As a cancer-related pathway, PI3K-Akt pathway has 18 cancer-related differentially expressed genes. They are listed at the bottom. Information about cancer-related genes in PI3K-Akt pathway was obtained from Networks of Cancer Genes (NCG 6.0) (http://ncg.kcl.ac.uk/index.php)

Next, we analyzed differentially expressed genes (DEG) among different genotypes [adjusted p value ≤ 0.05 and Fold Change(FC) ≥ 2]. WT cells were compared with each KO clone and each KO clone was compared with its corresponding rescue cells. Global visualization of fold changes of genes from each paired comparison is shown in volcano plots (Fig. 2A). Those DEGs are further displayed with heatmaps, which indicate that there are more genes downregulated than upregulated along with the loss of CREB1 (Fig. 2B). The same trend is also observed when we summarize the list of DEGs shared by all paired comparisons and with opposite expression profiles between CREB1 KO clones vs WT cells (KO vs WT) and CREB1 rescue cells vs CREB1 KO clones (Rescue vs KO) (Fig. 2C). Two exemplified genes, *BDKRB2* and *NCALD*, are labeled in the volcano plots as well (Fig. 2A). Consistently, between KO clones and WT cells, 225 genes are commonly downregulated while 107 genes are commonly upregulated (Additional file 1: Figure S1C). Within comparisons between CREB1 rescue cell lines and their corresponding KO clones, 443 genes are commonly upregulated while 108 genes are commonly downregulated (Additional file 1: Figure S1D). Collectively, our results suggest that more genes are positively regulated by CREB1.

To gain more insights into the enriched gene sets of the CREB1-regulated gene network, we summarized a CREB1 commonly regulated gene list and performed Gene Ontology (GO) analysis. This list contains genes showing similar differential expression patterns in at least two pairs of rescue clone vs its corresponding KO clone (Additional file 3: Table S2). The top ten biological processes were identified in upregulated genes and downregulated genes respectively (Fig. 2D). It shows that the CREB1-mediated gene network mainly promotes the regulation of transcription and signal transduction. Furthermore, we applied KEGG (Kyoto Encyclopedia of Genes and Genomes) pathway enrichment analysis, which provided us a detailed dissection of the CREB1-regulated signaling pathways. From the top upregulated ones, cAMP signaling pathway was significantly enriched even though none of our deep-sequenced cell lines had been treated with cAMP agonist (Fig. 2E). This might be due to the upregulated calcium signaling pathway (Fig. 2E), as subsequent occurrences of calcium activation and cAMP activation have been observed in cancer cells treated by cationic amphiphilic drugs [29]. Another interesting observation is that the PI3K-Akt signaling pathway was enriched in both upregulated and downregulated genes (Fig. 2E). Out of 47 DEGs from the PI3K-Akt signaling pathway, 12 cancer-relevant genes are upregulated while 6 are downregulated. Thus, it is more likely that CREB1 promotes the PI3K-Akt pathway in cancer cells. This inference is also supported by the fact that the aberrant activation of the PI3K-Akt pathway and overexpression of CREB1 occurs simultaneously to promote the survival and proliferation in many human cancer cells [2, 30].

### Cancer relevance determined by RRHO analysis

**Fig. 3:**
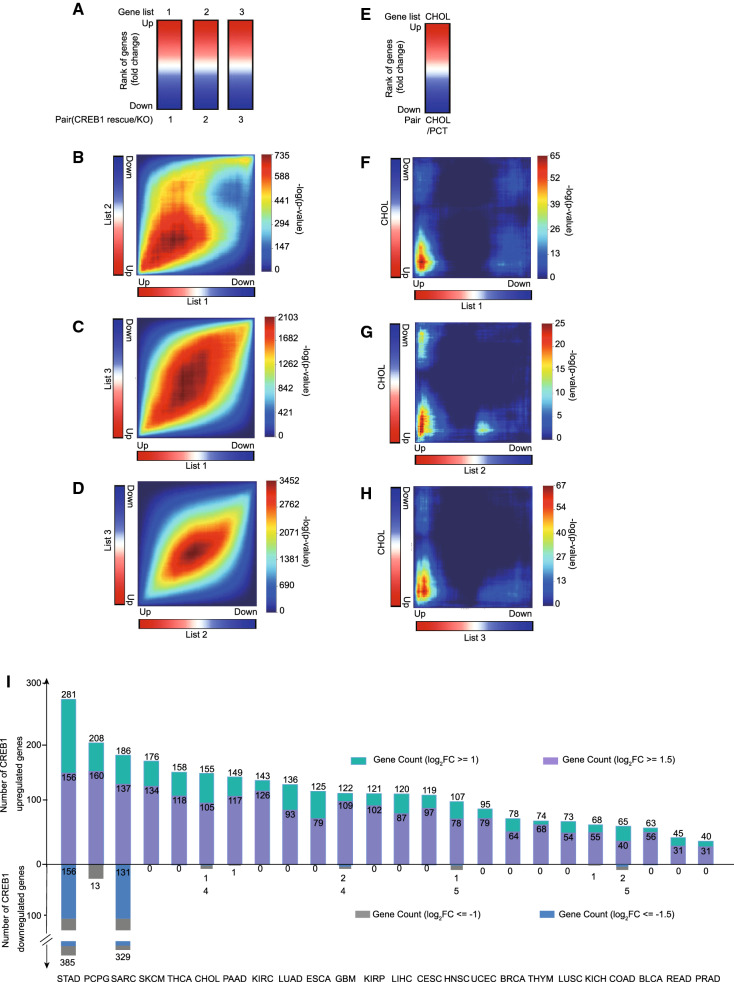
Rank-rank analysis of CREB1 rescue vs CREB1 KO gene ranks and tumor vs para-carcinoma tissue gene ranks. **A** Ranks of genes are displayed according to fold change of mRNA expression levels, which are obtained from the indicated pair-comparison analysis. Three lists of genes (Additional file 3: Table S2) from 3 pairs of Rescue and KO were generated with the indicated sequence. **B**–**D** Rank–rank hypergeometric overlap (RRHO) plots of lists 1 vs 2, 1 vs 3, and 2 vs 3. The plots are colored with -log transformed hypergeometric p value, representing the strength of overlap as positive and negative enrichment. **E** Rank of genes obtained from the pair-comparison analysis of mRNA expression levels in cholangiocarcinoma (CHOL) and paired para-carcinoma tissues (PCT). **F**–**H** Rank–rank hypergeometric overlap (RRHO) plots of lists CHOL vs 1, 2, 3 separately. Significantly overlapped genes (hypergeometric p value ≤ 0.05 of each comparison) from these 3 paired comparisons are selected. Those genes, showing similar overlap patterns in at least two pair-comparisons, are further added to the RRHO concordant gene list (Additional file 5: Table S4). The list contains both upregulated and downregulated genes. **I** Summary of no. of differentially expressed genes from the RRHO concordant gene list of 24 types of cancers. The threshold of average |log_2_ FC| was set bigger than either 1 or 1.5. The DEGs of 24 types of cancer and PCT comparison can be found in Additional file 4: Table S3. *BLCA* bladder urothelial carcinoma, *BRCA* breast invasive carcinoma, *CESC* cervical squamous cell carcinoma and endocervical adenocarcinoma, *COAD* colon adenocarcinoma, *ESCA* esophageal carcinoma, *GBM* glioblastoma multiforme, *HNSC* head and neck squamous cell carcinoma, *KICH* kidney chromophobe, *KIRC* kidney renal clear cell carcinoma, *KIRP* kidney renal papillary cell carcinoma, *LIHC* liver hepatocellular carcinoma, *LUAD* lung adenocarcinoma, *LUSC* lung squamous cell carcinoma, *PAAD* pancreatic adenocarcinoma, *PCPG* pheochromocytoma and paraganglioma, *PRAD* prostate adenocarcinoma, *READ* rectum adenocarcinoma), *SARC* sarcoma, *SKCM* skin cutaneous melanoma, *STAD* stomach adenocarcinoma, *THCA* thyroid carcinoma, *THYM* thymoma, *UCEC* uterine corpus endometrial carcinoma

To determine the cancer relevance of CREB1 commonly regulated genes, we sought to compare the gene expression change caused by the presence of CREB1 and the gene expression change between tumor and control (ctrl) tissue. This analysis requires paired normal and tumor tissue data from the same patient. We thus downloaded TCGA RNA-seq data for patients of 24 types of cancer, for which such paired data was available. For each type of cancer, we ranked genes according to the tumor vs. ctrl. tissue RNA-seq log_2_FC (Fig. 3E, Additional file 4: Table S3, Additional file 6: Table S5). Employing a threshold-free algorithm, Rank-rank Hypergeometric Overlap (RRHO) [31, 32], we made a genome-wide expression profile comparison among 3 pairs of Rescue vs KO or between Rescue vs KO and tumor vs ctrl. tissue. As the 3 pairs of Rescue vs KO are experimental repeats, it is reasonable that their mutual overlap patterns resemble good overlap with the most significant (red) area across the bottom left to top right diagonal of the map (Fig. 3A–D). In contrast, in the case of cholangiocarcinoma (CHOL), the overlap pattern between the two expression profiles shows that the most significant area is the bottom left. This demonstrates that upregulated gene lists in the two ranks share more in common (Fig. 3F–H), which implies that CREB1 upregulated genes tend to be overexpressed in CHOL. All the overlapped genes shared by at least two paired comparisons between Rescue vs KO and tumor vs ctrl. tissue are included in the RRHO concordant gene list. A similar analysis was applied to other types of cancer (Additional file 1: Figure S2). Eventually, we summarized the number of DEG (thresholds of both log_2_FC  ≥  1 and 1.5) from the RRHO concordant gene list of 24 types of cancer (Fig. 3I, Additional file 5: Table S4). Interestingly, unlike most types of cancer, in which CREB1 commonly regulated genes are mainly upregulated, stomach adenocarcinoma (STAD) and sarcoma (SARC) upregulate and downregulate CREB1 commonly regulated genes on a comparable scale (Fig. 3I). This suggests that there are both diversity and commonality in the CREB1-regulated gene network across different cancer types.

### Defining and categorizing CREB1-regulated genes by integrating transcriptomic, RRHO, and Chipseq analysis

**Fig. 4:**
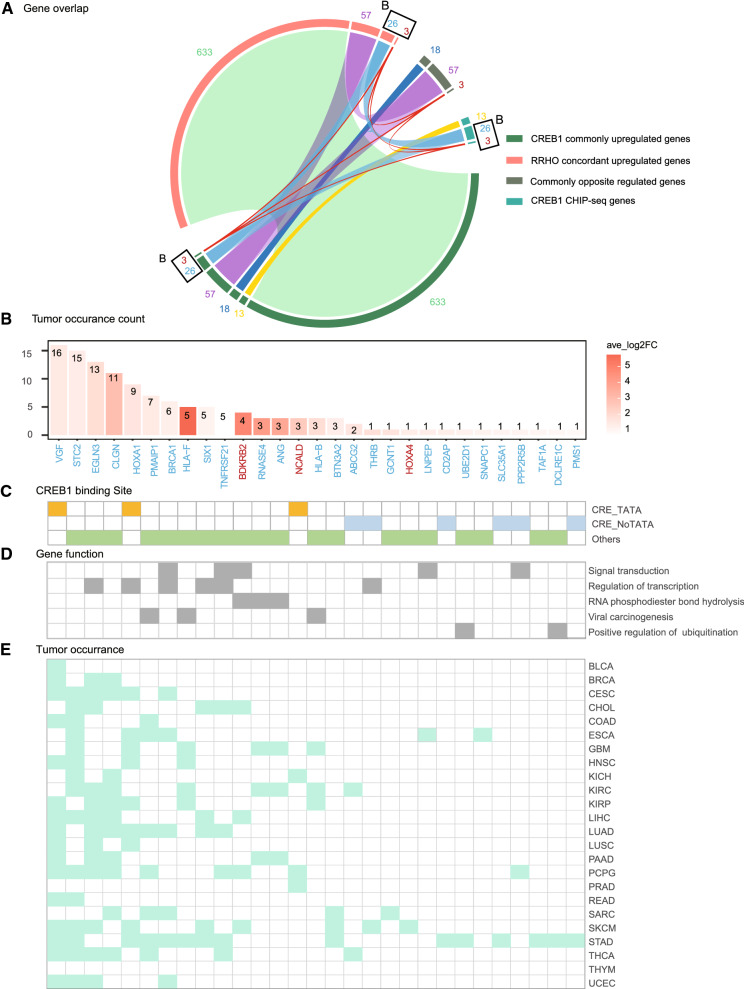
Integrative analysis of CREB1-upregulated gene network. **A** Gene distribution analysis of four different gene lists (R circlize package). They are (i) CREB1 commonly upregulated gene list; (ii) RRHO concordantly upregulated gene list; (iii) commonly opposite regulated genes between CREB1 rescue cells vs corresponding KO clones and KO clones vs WT cells; (iv) CREB1 CHIP-seq gene list (genes with p value ≤ 0.001, CREB1 binding ratio ≥ 2 are selected). The stripes of different colors connect the portions uniquely shared by different combinations of the lists (i–iv) (Additional file 5: Table S4). Three genes (red stripe) are present in all 4 lists. Twenty six genes (blue stripe) are uniquely shared by i, ii and iv. **B** 29 top-ranked CREB1-upregulated targets illustrated by their average log_2_ FC (Rescue/KO) and the tumor occurrence number in RRHO concordant gene list. The scale of log_2_ FC is displayed by the tomato gradient bar. **C**, **D** CREB1 binding sites in CHIP-seq data, gene function annotation based on GO analysis and detailed tumor occurrence of the 29 overlapped genes

Since a group of CREB1 target genes are transcription factors [7], many differentially expressed genes in CREB1 KO cells compared to WT cells or rescue cells might be indirectly regulated by CREB1. To further define and categorize CREB1-regulated genes, we sought to integrate transcriptomic, RRHO, and Chipseq analysis and made an intersection analysis of 4 lists of genes: (1) CREB1 commonly upregulated/downregulated genes (Rescue vs KO), (2) RRHO concordantly upregulated/downregulated genes, (3) Commonly opposite regulated genes (genes with opposite differential expression patterns between KO vs WT and Rescue vs KO in at least two paired comparisons), (4) CREB1 binding genes identified by Chipseq (Fig. 4A). The list of CREB1 binding genes was obtained from the previous database [7]. It provides us not only an important filter to distinguish CREB1 directly and indirectly regulated genes, but also the information about binding sequences. To identify CREB1 target genes with potential cancer relevance, we combined genes exclusively intersected by all 4 lists and by lists 1, 2, 4 as the top-ranked genes (Fig. 4B).

Our analysis obtained 29 top-ranked upregulated genes, which are further displayed with no. of tumor occurrence and their average expression fold change of CREB1 Rescue vs KO (Fig. 4B, D). Moreover, the characteristic of promoters of these top-ranked upregulated genes was summarized in Fig. 4C. Importantly, only three genes (*VGF, HOXA1, NCALD*) harbor a CRE-TATA motif in their promoters. This is consistent with our original hypothesis that CREB1 might constitutively regulate a set of genes independent of cAMP response. These top-ranked upregulated genes are mainly involved in signal transduction, regulation of transcription, RNA phosphodiester bond hydrolysis, viral carcinogenesis, and positive regulation of ubiquitination (Fig. 4D). To better describe these cancer-relevant CREB1 targeted genes, we further included intersection, fold change (CREB1 rescue cells vs corresponding CREB1 KO clone), and NO. of tumor occurrence into the ranking and generated a new rank list showed in Additional file 7: Table S6, in which *BRCA1* is the only confirmed CREB1 target gene [33] and there are 12 genes ranked higher than the *BRCA1*. Meanwhile, we chose the top upregulated gene, *BDKRB2*, to check its protein level accordingly. Compared to WT cells, BDKRB2 protein was greatly reduced in CREB1 KO cells, and this phenotype was reversed in CREB1 Rescue cells, further confirming *BDKRB2* as a novel CREB1 target gene (Additional file 1: Figure S5).

In parallel, we identified 20 top-ranked downregulated genes in total (Additional file 1: Figure S3A). Their average expression fold change of CREB1 Rescue vs KO, NO. of tumor occurrence, and promoters’ characteristics are displayed (Additional file 1: Figure S3B, C). Akin to top-ranked upregulated genes, the majority of top-ranked downregulated genes are independent of cAMP response. Moreover, they are mainly involved in cGMP-PKG signaling pathway, metabolic pathways, and positive regulation of epithelial cell proliferation. Interestingly, most of these genes, except *SEMA6A *which is also downregulated in kidney renal papillary cell carcinoma (KIRP), are exclusively downregulated in SARC and STAD (Additional file 1: Figure S3E).

Altogether, we conclude that CREB1 regulates expressions of targets positively in most cancer types except SARC and STAD, in which CREB1 appears to upregulate and downregulate distinct sets of genes simultaneously.

### Further validation of the significant cancer relevance of CREB1 target genes by Kaplan–Meier analysis

**Fig. 5:**
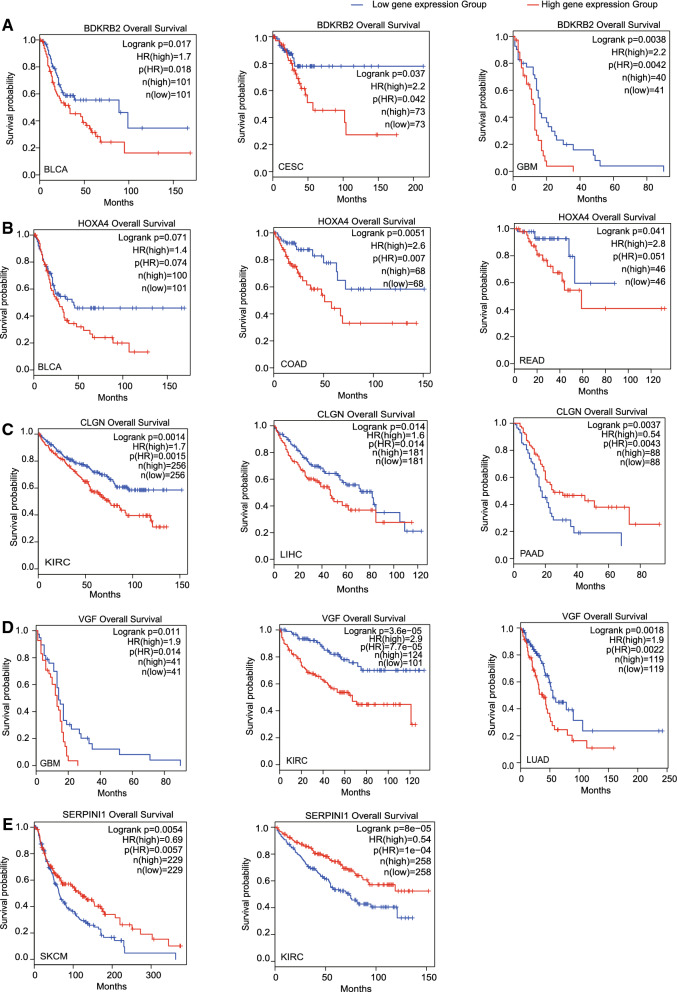
Cancer patient survival analysis of representative top-ranked CREB1 target genes. **A**–**D** Kaplan–Meier overall survival curves of top-ranked CREB1-upregulated targets (*BDKRB2*, *HOXA4*,* CLGN*, *VGF*) in different cancer types. **E** Kaplan–Meier overall survival curve of a top-ranked CREB1-downregulated target, *SERPINI1*, in different cancer types. log rank p value ≤ 0.05 was considered significant. Hazard ratio (HR) ≥ 1 and p (HR)  <  0.05 were considered significantly poor prognosis. n (high) and n (low) are the patient number

Lastly, to evaluate the influence of these top-ranked CREB1 target genes over cancer development, we performed Kaplan–Meier analysis of cancer patient's overall survival. For each gene, patients were divided into a high expression group and a low expression group according to their RNAseq data from TCGA database. In this manner, overall survival curves of the two groups were plotted accordingly. We selected *BDKRB2*, *CLGN*, *VGF*, *STC2*, *EGLN3*, *HOXA4 *as representative CREB1-upregulated targets and *SERPINI1*, *SLC22A4* as representative CREB1-downregulated targets. Our results showed that CREB1-upregulated targets are associated with poor prognosis while expressions of CREB1-downregulated targets are associated with improved prognosis (Fig. 5 and Additional file 1: Figure S4). Hazard ratios derived from Kaplan–Meier curves would further estimate the risk of patients’ death over the entire trial period. Consistently, hazard ratios of CREB1-upregulated targets are significantly higher than 1 while hazard ratios of CREB1-downregulated targets are significantly lower than 1. Interestingly, among the aforementioned genes, only *VGF*’s promoter has both TATA box and CRE, implying all others are cAMP irresponsive.

Furthermore, we reviewed the literature about top-ranked CREB1 targets. Many of their aberrant expressions are associated with angiogenesis, cell survival, resistance to cancer therapy, migration, etc. For example, as top-ranked CREB1-upregulated targets, *STC2* and *ABCG2* promote proliferation and metastasis of pancreatic cancer cells and hepatocellular carcinoma respectively [34, 35], while as top-ranked CREB1-downregulated targets,* SERPINI1* and *SEMA6A* are lowly expressed and fail to exert tumor-suppressive effects in gastric and lung cancer cells respectively [36, 37]. Moreover, some of these CREB1 targets are regarded as prognostic indicators and biomarkers for distinct types of cancer. For example, *BDKRB2*, the NO.1 CREB1-upregulated target, is the receptor of Bradykinin that is a diagnostic marker for cervical cancer [38]. Overexpression of *SIX1*, another top-ranked CREB1-upregulated target, is an independent poor prognostic marker for stage I–III colorectal cancer [39]. In this regard, we summarize the information about all top-ranked CREB1 targets that are reported as cancer biomarkers (Table 1). To our knowledge, we are the first to establish the transcriptomic correlation between CREB1 and most of these essential cancer-relevant genes.

**Table 1:** Cancer biomarker information of top-ranked CREB1 targets

Gene	Up-/down-regulated	Oncogene (+)/tumor suppressor gene (−)	Biomarker/potential biomarker
BDKRB2	↑	+	Cervical cancer [38]
HLA-F	↑	+	Breast cancer [40]; glioma [41]
VGF	↑	+	Pulmonary neuroendocrine tumors [42]; urothelial cell carcinoma [43]
STC2	↑	+	Esophageal squamous cell carcinoma [44]; renal cell carcinoma [45]; colorectal cancer [46]
NCALD	↑	+	Cytogenetic normal acute myeloid leukemia [47]; ovarian cancer [48]
BRCA1	↑	−	Sporadic epithelial ovarian carcinoma [49]; breast cancer[50]
HOXA4	↑	−	Lung cancer [51]
SIX1	↑	+	Stage I–III colorectal cancer [39]; cervical cancer [52]; KRAS/LKB1-mutant lung cancer [53]
BTN3A2	↑	−	Triple-negative breast cancer [54]; pancreatic ductal [55]; adenocarcinoma [56]
ABCG2	↑	+	Cancer stem cell [57]
UBE2D1	↑	+	Lung adenocarcinoma [58]
TAF1A	↑	+	Cervical cancer [59]
EPHA7	↓	−	Immune checkpoint inhibitors in multiple cancers [60]
USP11	↓	+	Colorectal cancer [60, 61]; breast cancer [62]
RBM3	↓	−	Head and neck squamous cell carcinoma [63]

Taken together, the above data strongly suggests that CREB1 constitutively regulates a set of its target genes to promote tumorigenesis.

## Conclusions

CREB1 is a multi-functional transcription factor whose activity is tightly regulated by the level of the intracellular cAMP. Due to its physiological importance, the CREB1-regulated gene network in response to increased cAMP has been extensively studied, particularly in normal neuronal tissues and metabolic tissues [64–67]. The genome-wide analysis revealed that the cAMP responsiveness of CREB1 target genes requires a simultaneous presence of TATA box and CRE in promoters [6]. However, among more than 4000 Chipseq-identified CREB1 target genes, only 339 genes meet this criterion, and even less than 100 genes are validated. This may be partially due to the conditional recruitment of the coactivator proteins to those promoters in different physiological contexts. For example, in 293 T cells, induction of CREB1 target genes by cAMP requires the recruitment of both CREB1 and CBP to the promoters [7]. In another instance, CREB1 associates with CRTC2 to activate autophagy genes in fasting mice while it associates with *FXR* to suppress autophagy genes in fed mice [66]. Besides, there exists another possibility, that is CREB1 can constitutively regulate a set of target genes independent of cAMP response. This might be true, particularly in the context of tumors, as only a few CREB1 target genes essential for cancer cell survival, proliferation and metastasis have been identified and most of them are regulated in this manner. Most importantly, compelling evidence demonstrates that CREB1 plays an essential role in promoting tumor development. So a comprehensive and in-depth analysis of the gene network constitutively regulated by CREB1 can be necessary and important for understanding how CREB1 functions in cancer cells and providing a theoretical basis for tailored cancer therapies.

Prompt by this, we firstly generated CREB1 KO cervical cancer cells and corresponding rescue cells. Transcriptomic analysis of these cells without any cAMP agonists enabled us to obtain a CREB1 commonly regulated gene list, which was further processed to gain general knowledge about the signaling pathways controlled by CREB1. Moreover, we extract a CREB1 concordantly regulated gene list by applying RRHO analysis with both our data and RNAseq data of cancer patients from TCGA. This list summarized the genes upregulated/downregulated in both CREB1 rescue cells compared to CREB1 KO cells and patients’ tumors compared to adjacent normal tissues. Importantly, the RRHO analysis patterns also demonstrate that there is a significant rank overlap between some CREB1-upregulated genes and highly expressed genes in most types of tumors. As CREB1 overexpression was found in many tumor types [68], it is very likely that CREB1 is mainly responsible for controlling these CREB1 concordantly regulated genes in cancer cells. To pinpoint CREB1 direct target genes with strong cancer relevance, we brought previous CREB1 Chipseq data into our analysis. After a mutual exclusive intersection analysis of four lists (CREB1 commonly upregulated/downregulated genes, RRHO concordantly upregulated/downregulated genes, commonly opposite regulated genes, and CREB1 Chipseq genes), we got 29 top-ranked CREB1-upregulated targets and 20 top-ranked CREB1-downregulated targets. Because of CREB1’s role as a proto-oncogene, it is presumable that the top-ranked CREB1-upregulated targets are prone to promote tumors while the top-ranked CREB1-downregulated targets are prone to suppress tumors. We affirmed this inference by comparing the overall survival and hazard ratios of cancer patients with high and low expression of selected genes from the top-ranked CREB1 target list, meaning that our methodology is very reliable. Interestingly, most of these genes have no simultaneous presence of TATA box and CRE in their promoters, implying that they are cAMP irresponsive. More importantly, after reviewing the functions of these genes, many of their aberrant expressions are found to be associated with more aggressive characteristics of cancers. This is in agreement with our initial hypothesis, that CREB1 constitutively regulates a set of targets to promote cancer development in a cAMP-independent manner.

Our systematic and integrative approach to decipher the CREB1-mediated gene network in cancer is an effective and powerful tool that can be applied in other transcription factor-related research. This method can substantially shorten the studies with similar purposes which usually take 3–10 years. The major limitation is that our approach relies on resourceful expression databases derived from patients samples. The more stratified expression databases are, the more informative our approach turns out to be. Thus, in terms of cancer research, RNAseq databases with more details about tumor stages, molecular subtypes, and therapeutic outcomes are preferred, especially for a prognosis purpose. Moreover, to verify any transcription factor-regulated biomarkers for diagnosis and prognosis, more solid preclinical and clinical studies are still of necessity. Since our approach can narrow down the scope of candidate genes in a faster way, its application will greatly promote the identification of novel biomarkers for any disease with resourceful expression databases.

Taken together, our current work highlights the importance of the gene network constitutively regulated by CREB1 in cancer cells. Moreover, the whole in-depth bioinformatics analysis provides a valuable and comprehensive reference to any further investigation about CREB1 and its targets. Our methodology can be applied to study other transcription factors and investigate the disease relevance of their target genes.

## Materials and methods

### Reagents

Restriction enzymes were purchased from NEB (New England Biolabs Inc.). Oligonucleotides and primers were ordered from BGI-Qingdao (synthetic biology platform), China. In-Fusion HD Cloning Kit (#639649) and Premix ExTaq Version 2.0 (#D332C) were purchased from Takara. Dulbecco’s modified Eagle’s medium (DMEM #12430054), Pfx polymerase (#C11708-021) and GlutaMAX (#A12860-01) was purchased from Invitrogen. Fetal bovine serum (FBS #16000044), 1  ×  phosphate buffered saline (PBS #14190500BT), Opti-MEM (#31985062), Penicillin–Streptomycin (#15140122) and 0.25% Trypsin–EDTA (#25200072) were purchased from Gibco. PEI 40,000 (#24765-1) and PEG-itTM (#LV810A-1) Virus Precipitation Solution were purchased from Polysciences and System Biosciences respectively. Protamine sulfate (#P3369-10G), and proteinase K (#P6556) were purchased from Sigma-Aldrich. Puromycin (#58-58-2) and hygromycin B (#97064-454) was purchased from InvivoGen and VWR respectively. Lipofectamine 3000 transfection agents (# L3000015), NP40 (#FNN0021) Trizol solution (#15596026) and KCl (#AM9640G) were purchased from Thermo Fisher. Cell Lysate Buffer (#P0013), Pierce Bicinchoninic acid (BCA) Protein Assay Kit (#P0011), 1 M Tris–Hcl pH7.6 (#ST776), Primary and Secondary Antibody Dilution Buffer (#P0023A, #P0023D) and SDS-PAGE Protein Staining and Loading Buffer (5X) (#P0280) for Western Blot were purchased from Beyotime. MgCl2 (#20–303), ECL WB Detection Kit (#GERPN2232) and PVDF membranes (# IPVH00010) were purchased from Merck. Tween-20 (#DH358-2) was purchased from Dingguo, China. Non-fat powdered milk (#A600669-0250) and Tris (#0497-500 g) were purchased from BBI Life Science.

### Cell culture

HeLa cervix carcinoma (HeLa) cells were gift from Ocean University of China and cultured in DMEM supplemented with 10% FBS, 1% GlutaMAX at 37 °C with 5% CO_2_ atmosphere and maximum humidity. They were passaged every 1–2 days at 1:4 ratio. This process was done by gently washing cells twice with equal volume as growth medium of 1  ×  PBS without calcium and magnesium, followed by cell detachment by 0.25% Trypsin–EDTA for 3 min at 37 °C. HeLa cells were used within 6 months after thawing.

### Plasmids

Lentivirus packaging plasmids including PSRV-REV, PMD.2G and PMDGp-lg/p-RRE were gifts from Biomedicine department in Aarhus University, Denmark. The pMAX-GFP (VDF-1012) plasmid was supplied by the Amaxa nucleofection kit (#VPI-1003, Lonza) and used as a fluorescence control plasmid during transfection.

Two sgRNAs targeting the upstream and downstream sequences of CREB1 exon 1 (including TATA box area) were designed by the online software tool CRISPOR (http://crispor.tefor.net). Accordingly, two pairs of oligos (SS1: CACCGCACCTCTCCTTGTTAGTAG, AS1: AAACCTACTAACAAGGAGAGGTGC; SS2: AAACCTACTAACAAGGAGAGGTGC, AS2: AAACTATCTGAGAGCACCATTTAC) were annealed and ligated into lentiCRISPRv2 vector (Addgene plasmid #52961) at BamHI site according to the optimized golden gate assembly protocol [69]. The resulting plasmids were named as lenti CREB1SgRNA1 and lenti CREB1SgRNA2.

CREB1 cDNA was cloned into lentiCRISPRv2 via XbaI and BamHI sites, by which the Cas9 cDNA was replaced. Thereafter, hygromycin-resistance gene driven by EF1 promoter from PB-TRE-dCas9-VPR plasmid (Addgene plasmid #63800) was further introduced into the above plasmid using In-Fusion HD Cloning Kit. The resulting plasmid is named as lentiCREB1hygro.

### Generation of HeLa CREB1 KO cells

The efficacy of two sgRNAs targeting CREB1 were examined and confirmed by Ccheck system[70]. HeLa cells were co-transfected with lenti CREB1SgRNA1 and lenti CREB1SgRNA2 by Lipofectamine 3000 according to the manufacture’s instruction. Potential knockout clones were selected by culturing the transfected cells in a growth medium supplemented with puromycin (1 μg/mL) for 3 weeks. Single-cell clones were manually picked under a stereomicroscope and cultured in 96-well plates until 70–80% confluence. Thereafter, authentic clones carrying homozygous deletion of CREB1 exon 1 were verified by PCR screening and sanger sequencing (forward primer: GGCAACAGAGCAAGACCTCA; Reverse primer: AGTTGAGGAGAATGCATGCA) after they were lysed by cell lysis buffer (KCl 50 mM, MgCl2 1.5 mM, 0.5% NP40 and 0.5% Tween 20, 10 mM Tris pH 8.5) [71].

### Generation of HeLa CREB1 rescue cells

LentiCREB1hygro and 3 lentivirus packaging plasmids were co-transfected into HEK293T cells by PEI 40,000 transfection reagent according to the manufacture’s instruction. 48 h later, viral particles were collected and precipitated by PEG-itTM Virus Precipitation Solution according to the manufacture’s instruction. Thereafter, CREB1 KO cells growing on a 6-well plate were infected in virus-containing medium supplemented with 8 μg/ml protamine sulfate through spin-infection (2400  ×  g for 90 min). After another 48 h, infected cells were transferred to 10 cm petri-dishes with growth medium supplemented with 200 μg/mL hygromycin B. Finally, CREB1 rescue cell lines were obtained by 3-weeks selection in hygromycin-containing medium. Western Blots were performed to confirm the success of infections and re-expression of CREB1 in each corresponding CREB1 KO clones.

### Western Blot analysis

Cells were lysed by Western Blot Cell Lysate Buffer according to the manufacture’s instruction. Protein concentrations of cell lysates were determined by using BCA Protein Assay Kit. After denaturing proteins by heat-inactivation in loading buffer, each sample was loaded on SDS polyacrylamide gels and separated by electrophoresis. Thereafter, proteins were transferred to PVDF membranes by using Bio-Rad Trans-Blot Turbo system. Subsequently, membranes were blocked with Tris-buffered saline buffer containing 5% non-fat milk and 0.1% Tween-20 at 4 °C overnight. Then membranes were incubated with primary antibodies (CREB1 #9197, cell signaling, and anti-LaminB1 #ab194109, Abcam) and anti-rabbit peroxidase-conjugated secondary antibody (#7074, cell signaling) stepwise. Between any steps, membranes were washed by TBS–Tween buffer extensively. Finally, the signal was detected with ECL WB Detection Kit by Azure600 Western Blot Imaging System (Azure Biosystems).

### RNA-seq analysis

HeLa cells were detached from 10 cm petri-dishes and total RNA was isolated and separated from DNA and protein after extraction with Trizol solution. RNA-library preparation and sequencing were done by BGI-Qingdao, producing 100 bp paired-end non-stranded libraries with  ~  30 M reads per library using BGI-500 sequencer. 21 libraries were prepared, including clones CREB1 KO1, CREB1 KO2, CREB1 KO3, CREB1 rescue1, CREB1 rescue2, CREB1 rescue3 and WT, and each sample had 3 replicates. RNA sequencing results can be found in the Additional file 2: Table S1. RNA-seq reads were aligned to human genome (GRCh37/hg19) using Bowtie2 [72] within FASTQ file with default settings after removing the low-quality and short reads with SOAPnuke v1.5.6 [73] (Additional file 2: Table S1). Unique mapped reads were kept for further analysis. The read counts were quantified by Expectation–Maximization (RSEM v1.3.2) [74], which were used for statistical analyses of differential expressed genes (DEGs) by using DESeq2 package [75] in R. The overall similarities between samples showed in the hierarchical samples clustering heatmaps and principal components analysis (PCA) plots (Additional file 1: Figure S1) were calculated by Euclidean sample distance and ordination method that come with DESeq2 package to visualize sample-to-sample distance. DEGs were defined as genes with a Benjamini-Hochberg–adjusted p-value (p-adj)  ≤  0.05 and log_2_ Fold Change (log_2_ FC)  ≥  1 in comparing CREB1 KO cells vs WT cells, and CREB1 rescue cells vs CREB1 KO cells (Additional file 3: Table S2).

### GO enrichment analysis and KEGG pathway analysis

Enrichment tests for GO (Gene Ontology) terms and KEGG (Kyoto Encyclopedia of Genes and Genomes) pathway analysis were performed on CREB1 commonly expressed gene list (Additional file 3: Table S2) that were selected as defined using David website (david.ncifcrf.gov). The background gene set was set to Homo species. The significance threshold was set to Benjiamini-Hochberg false discovery rate (FDR)  ≤  0.05 for KEGG pathway analysis and  ≤  0.01 for GO enrichment analysis.

### RRHO analysis

All expressed genes from RNA-seq experiments in three CREB1 rescue cell lines vs corresponding CREB1 KO cell lines were ranked according to fold change value. For every type of cancer, genes expressed both in carcinomas and para-carcinoma tissues (PCT) were ranked according to fold change value (Additional file 4: Table S3). The paired CREB1 rescue/KO ranked gene lists were compared to paired cancer/PCT ranked gene lists of 24 types of cancer from Cancer Genome Atlas (TCGA) (https://www.cancer.gov/tcga) using Rank-Rank Hypergeometric Overlap (RRHO) function (https://bioconductor.org/packages/RRHO) [31] in R. Heatmaps were obtained from each comparison, showing rank-rank correlations. The gradient color was calculated by log_10_-transformed hypergeometric p-value of local rank overlaps between gene lists. 1362 overlap genes (719 upregulated and 643 downregulated DEGS in CREB1 rescue/KO comparison) were selected to RRHO concordant gene list (Additional file 5: Table S4).

### Patient survival analysis

Kaplan–Meier analysis was performed to estimate the overall survival (OS) of patients on online tool GEPIA2 (http://gepia2.cancer-pku.cn/#survival). Patients were categorized into high and low gene expression groups according to the cut-off value determined either by median or quartile gene expression. p values were calculated using the log-rank test and considered statistically significant when p value  ≤  0.05.

### Statistical analysis

DESeq2 and RRHO analyses were performed in R (4.0.2). For identification of DEGs, we used an adjusted p value (Padj)  ≤  0.05 and fold change (FC)  ≤  2 as cut-off. GO terms with p ≤  0.01 were included.

## Supplementary Information

**Additional file 1: Figure S1.** RNA-seq analysis of CREB1 KO cells, CREB1 rescue cells, and WT cells. A Hierarchical clustering heatmap showing overall similarity between samples based on Euclidean distance calculation between CREB1 KO vs WT and Rescue vs KO. B Principal component analysis (PCA) showing sample variance based on gene expression. Points that represent samples were projected onto the 2D plane. Sample variance was indicated by Variance Percent. C, D Summary of DEGs in paired comparison of CREB1 KO vs WT and Rescue vs KO respectively (R upset package). Side horizontal bars indicate the total number of DEGs of each paired comparison. Vertical bars indicate the total number of DEGs exclusively existed in indicated combinations of paired comparisons. **Figure S2.** Rank-rank analysis of CREB1 rescue vs CREB1 KO gene ranks and tumor vs para-carcinoma tissue gene ranks. Rank–rank hypergeometric overlap (RRHO) plots of lists of 24 types of cancer vs 1, 2, 3 separately. **Figure S3.** Integrative analysis of CREB1-downregulated gene network. A Gene distribution analysis of four different gene lists (R circlize package). They are (i) CREB1 commonly downregulated gene list; (ii) RRHO concordant downregulated gene list; (iii) commonly opposite regulated genes between Rescue vs KO and KO vs WT; (iv) CREB1 Chipseq gene list (genes with p value ≤ 0.001, CREB1 binding ratio ≥ 2 are selected). The stripes of different colors connect the portions uniquely shared by different combinations of the lists (i–iv) (Additional file 5: Table S4). 1 gene (red stripe) are present in all 4 lists. 19 genes (blue stripe) are uniquely shared by i, ii, and iv. B. 20 top-ranked CREB1-downregulated targets illustrated by their average log_2_ FC (CREB1 rescue vs CREB1 KO) and the tumor occurrence number in RRHO concordant gene list. The scale of log_2_ FC is displayed by the tomato gradient bar. C, D. CREB1 binding sites in Chipseq data, gene function annotation based on GO analysis and detailed tumor occurrence of the 20 overlapped genes. **Figure S4.** Cancer patient survival analysis of representative top-ranked CREB1 target genes. A, B Kaplan-Meier overall survival curves of top-ranked CREB1-upregulated targets (*STC2*, *EGLN3*) in different cancer types. C Kaplan-Meier overall survival curve of a top-ranked CREB1-downregulated target, *SLC22A4*, in different cancer types. log rank p value ≤ 0.05 was considered significant. Hazard ratio (HR) ≥ 1 and p (HR) < 0.05 were considered significantly poor prognosis. n (high) and n (low) are the patient number. **Figure S5.** Validation of *BDKRB2* as a novel CREB1-upregulated target by western blot. Western blot of indicated proteins in wild type (WT), CREB1 KO, and CREB1 rescue (R) cells. Due to a bipartite nuclear localization signal and a Flag tag at CREB1’s C-terminal, the molecular weight of CREB1 in CREB1 rescue cells is 8KD bigger than in WT cells.

**Additional file 2.**  RNA sequencing results.

**Additional file 3.** CREB1 differencial expressed gene list and CREB1 commonly expressed gene list.

**Additional file 4.** Differential expressed genes in carcinomas and para-carcinoma tissues (PCT) from TCGA.

**Additional file 5.** RRHO concordant gene list.

**Additional file 6.** TCGA case data of 24 types of cancer.

**Additional file 7.** CREB1 targeted gene list was ranked based on the number of tumor occurrence.

## Data Availability

All the RNAseq data are stored in CNGB database (https://db.cngb.org/). The accession number is CNP0001581. All data generated or analyzed during this study are included in this published article [and its supplementary information files].
