# Supplementary material for: Systematical analysis reveals a strong cancer relevance of CREB1-regulated genes
Source: Cancer Cell Int. 2021 Oct 12;21:530. doi: 10.1186/s12935-021-02224-z (PMC8507136; doi:10.1186/s12935-021-02224-z)

Figure S1

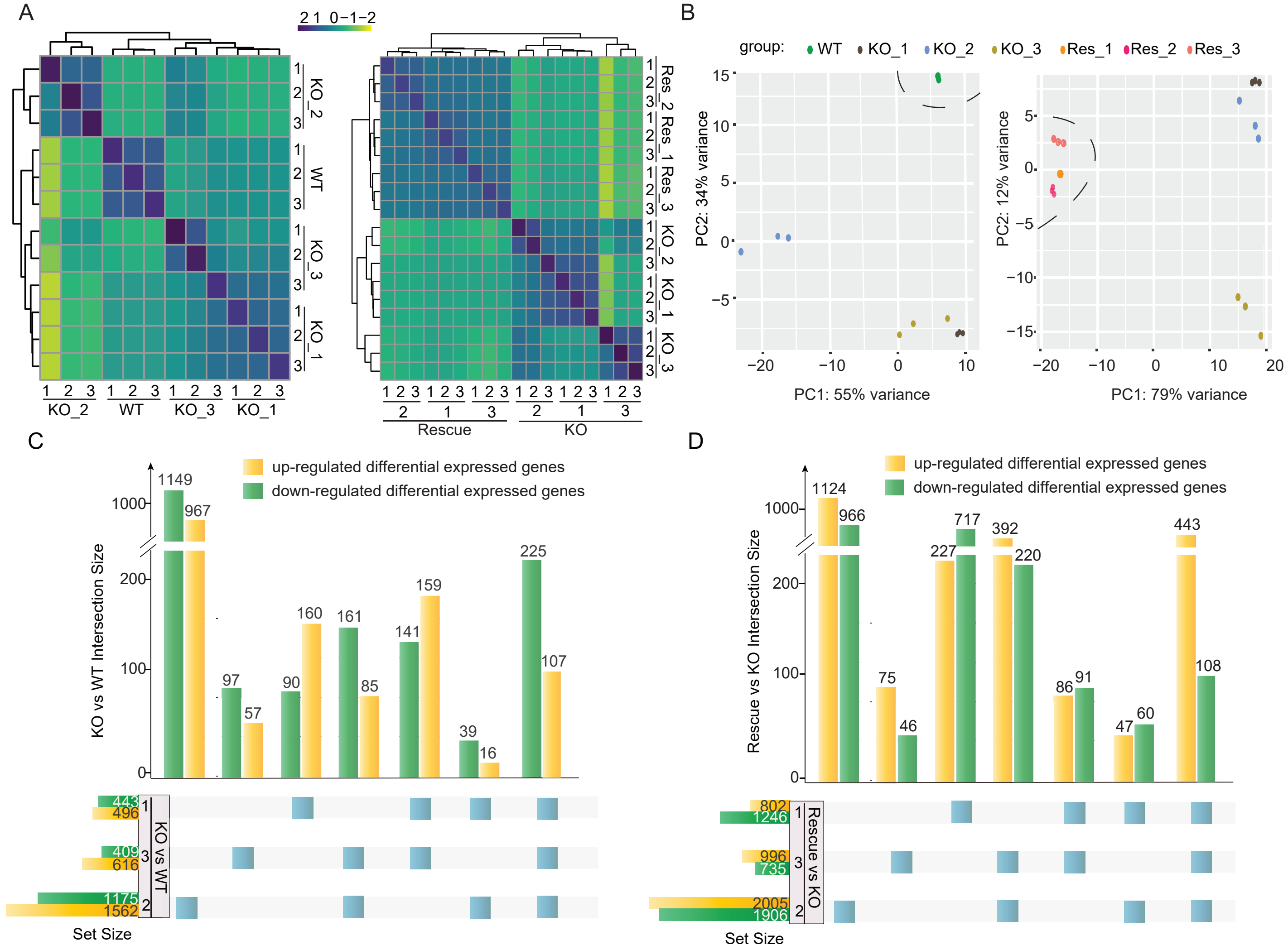

A
KO_2
WT
KO_3
KO_1
Rescue
KO
Res_2
Res_1
Res_3
B
group: WT KO_1 KO_2 KO_3 Res_1 Res_2 Res_3
PC1: 55% variance
PC2: 34% variance
PC1: 79% variance
PC2: 12% variance
C
up-regulated differential expressed genes
down-regulated differential expressed genes
KO vs WT Intersection Size
KO vs WT
Set Size
D
up-regulated differential expressed genes
down-regulated differential expressed genes
Rescue vs KO Intersection Size
Rescue vs KO
Set Size

Figure S2

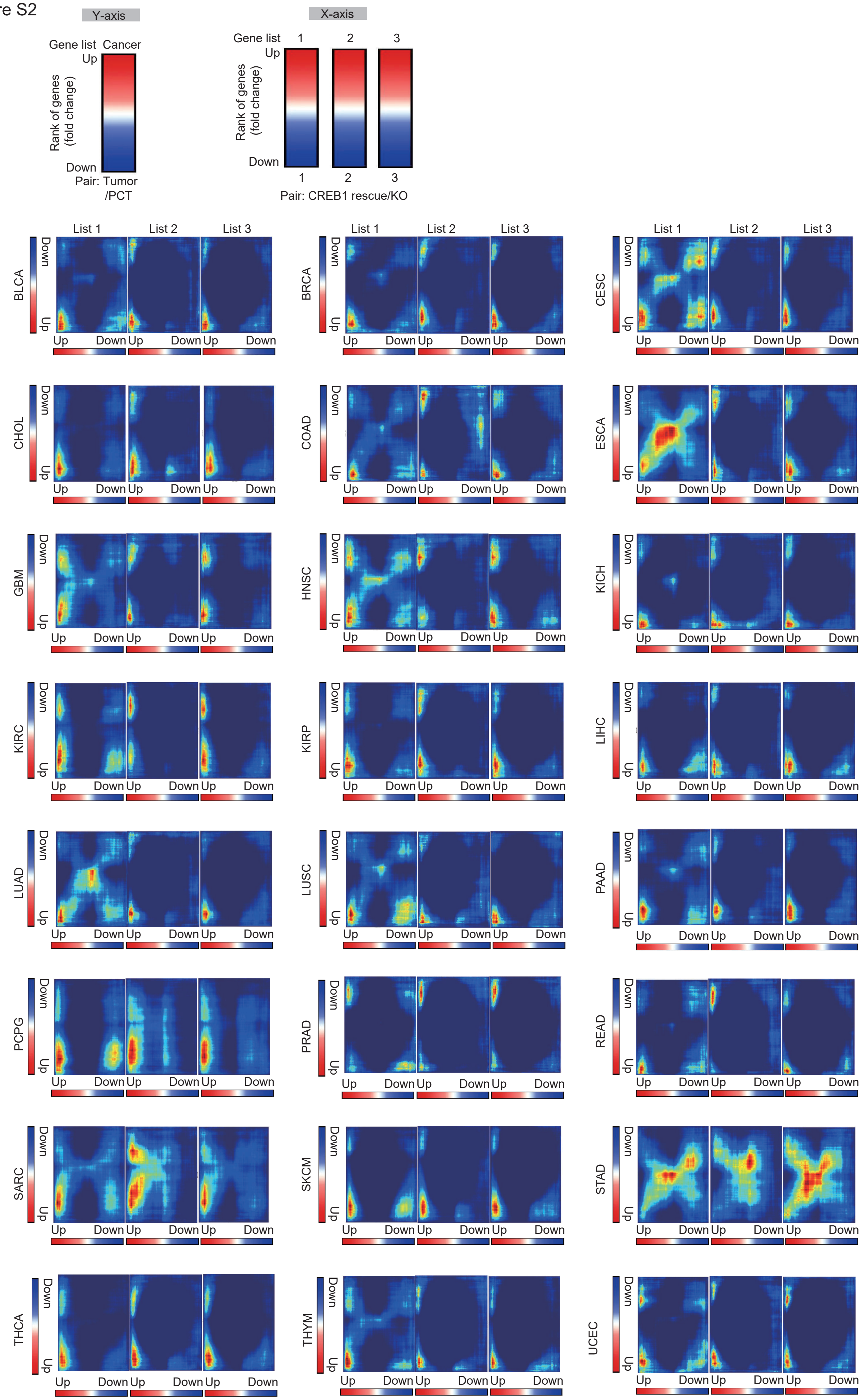

Y-axis
Gene list Cancer
Up
Rank of genes (fold change)
Down
Pair: Tumor /PCT
X-axis
Gene list 1 2 3
Up
Rank of genes (fold change)
Down
1 2 3
Pair: CREB1 rescue/KO
List 1 List 2 List 3
BLCA
BRCA
CESC
CHOL
COAD
ESCA
GBM
HNSC
KICH
KIRC
KIRP
LIHC
LUAD
LUSC
PAAD
PCPG
PRAD
READ
SARC
SKCM
STAD
THCA
THYM
UCEC
Up Down

Figure S3

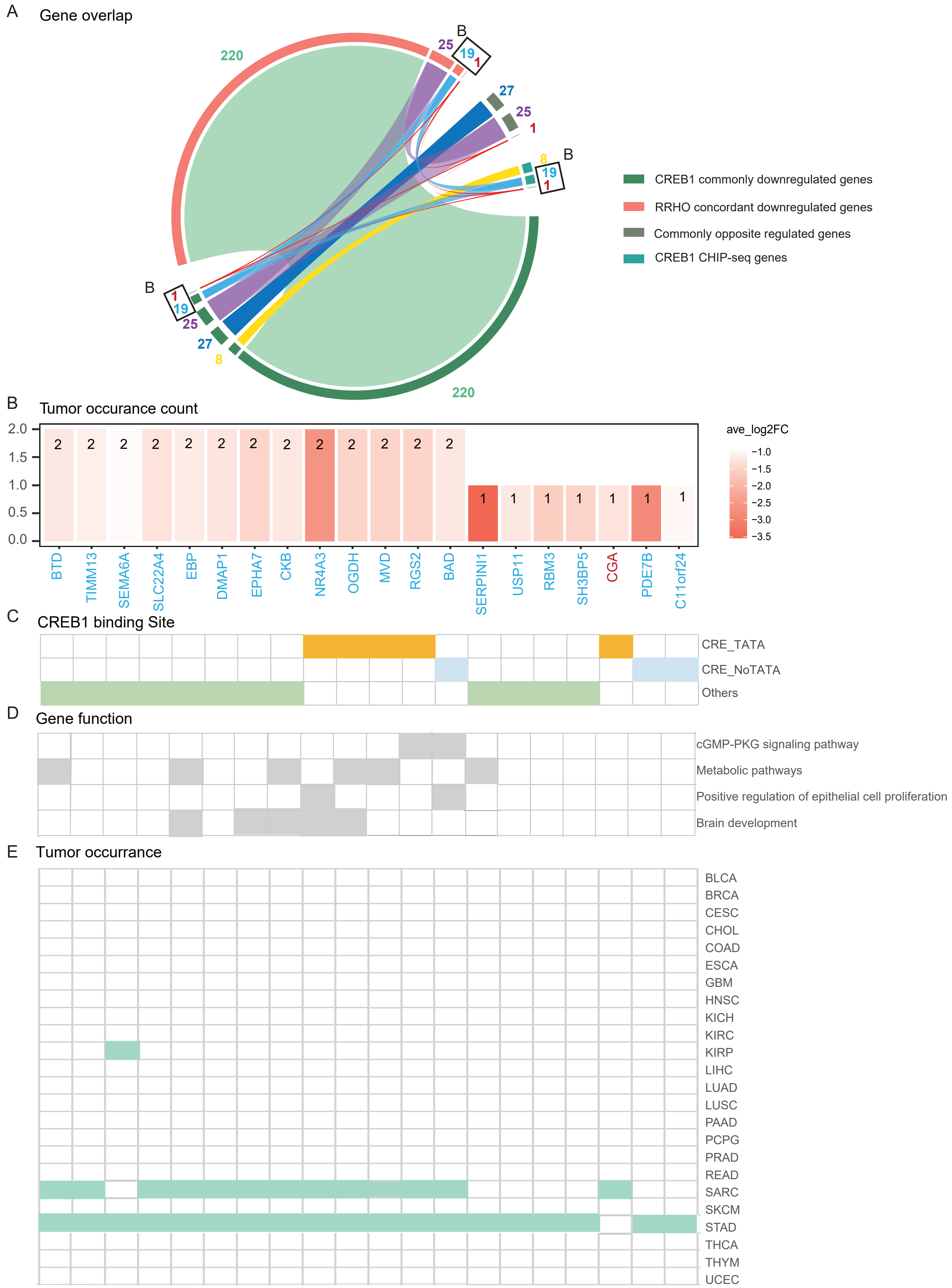
A
Gene overlap
220
25
19
1
B
27
25
1
8
19
1
B
B
1
19
25
27
8
220
CREB1 commonly downregulated genes
RRHO concordant downregulated genes
Commonly opposite regulated genes
CREB1 CHIP-seq genes
B
Tumor occurance count
ave_log2FC
−1.0
−1.5
−2.0
−2.5
−3.0
−3.5
2.0
1.5
1.0
0.5
0.0
BTD
TIMM13
SEMA6A
SLC22A4
EBP
DMAP1
EPHA7
CKB
NR4A3
OGDH
MVD
RGS2
BAD
SERPINI1
USP11
RBM3
SH3BP5
CGA
PDE7B
C11orf24
C
CREB1 binding Site
CRE_TATA
CRE_NoTATA
Others
D
Gene function
cGMP-PKG signaling pathway
Metabolic pathways
Positive regulation of epithelial cell proliferation
Brain development
E
Tumor occurrance
BLCA
BRCA
CESC
CHOL
COAD
ESCA
GBM
HNSC
KICH
KIRC
KIRP
LIHC
LUAD
LUSC
PAAD
PCPG
PRAD
READ
SARC
SKCM
STAD
THCA
THYM
UCEC

Figure S4

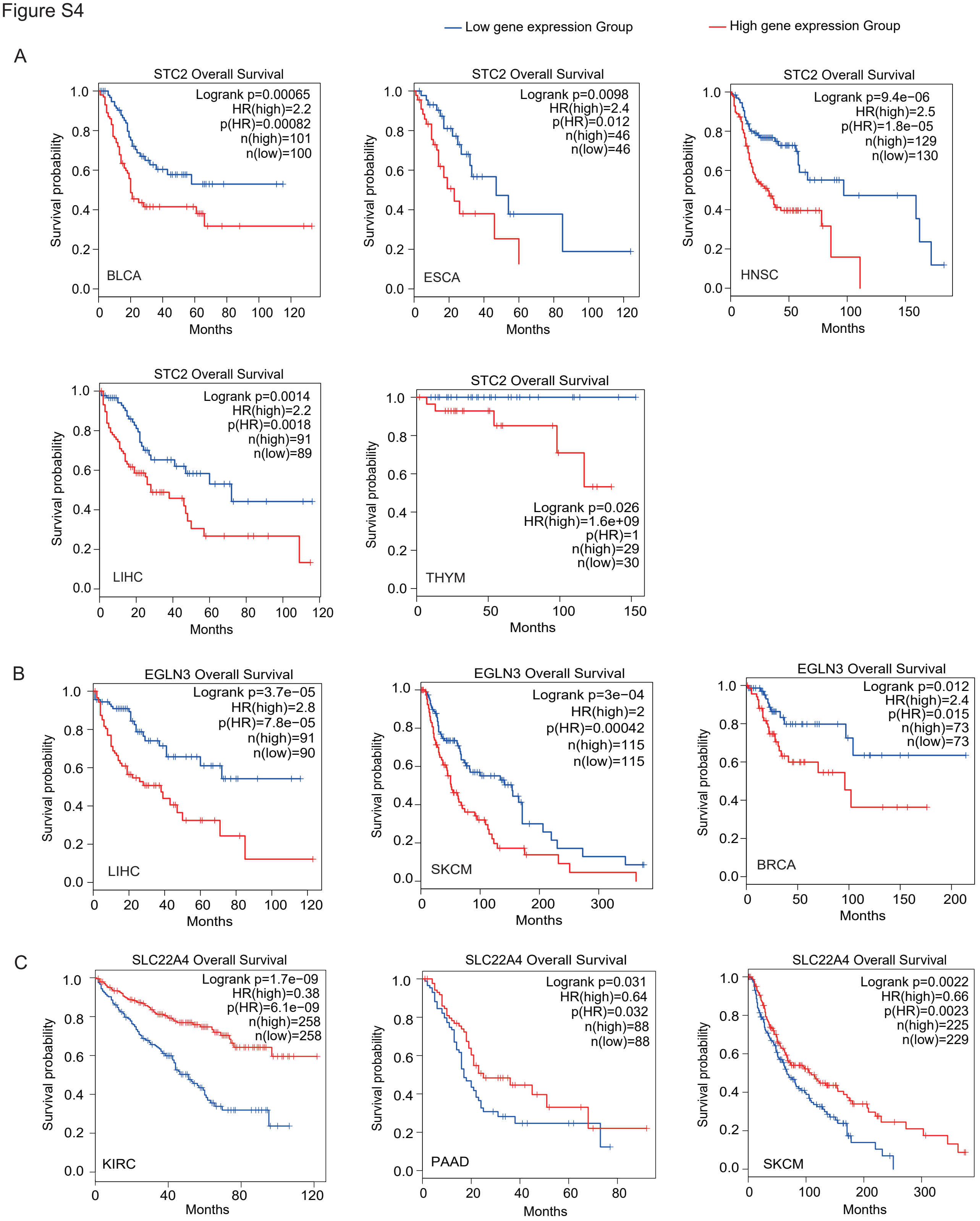

Low gene expression Group
High gene expression Group
A
STC2 Overall Survival
Logrank p=0.00065
HR(high)=2.2
p(HR)=0.00082
n(high)=101
n(low)=100
BLCA
STC2 Overall Survival
Logrank p=0.0098
HR(high)=2.4
p(HR)=0.012
n(high)=46
n(low)=46
ESCA
STC2 Overall Survival
Logrank p=9.4e−06
HR(high)=2.5
p(HR)=1.8e−05
n(high)=129
n(low)=130
HNSC
STC2 Overall Survival
Logrank p=0.0014
HR(high)=2.2
p(HR)=0.0018
n(high)=91
n(low)=89
LIHC
STC2 Overall Survival
Logrank p=0.026
HR(high)=1.6e+09
p(HR)=1
n(high)=29
n(low)=30
THYM
B
EGLN3 Overall Survival
Logrank p=3.7e−05
HR(high)=2.8
p(HR)=7.8e−05
n(high)=91
n(low)=90
LIHC
EGLN3 Overall Survival
Logrank p=3e−04
HR(high)=2
p(HR)=0.00042
n(high)=115
n(low)=115
SKCM
EGLN3 Overall Survival
Logrank p=0.012
HR(high)=2.4
p(HR)=0.015
n(high)=73
n(low)=73
BRCA
C
SLC22A4 Overall Survival
Logrank p=1.7e−09
HR(high)=0.38
p(HR)=6.1e−09
n(high)=258
n(low)=258
KIRC
SLC22A4 Overall Survival
Logrank p=0.031
HR(high)=0.64
p(HR)=0.032
n(high)=88
n(low)=88
PAAD
SLC22A4 Overall Survival
Logrank p=0.0022
HR(high)=0.66
p(HR)=0.0023
n(high)=225
n(low)=229
SKCM
Survival probability
Months

Figure S5

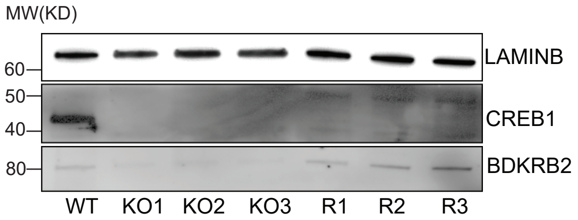

Supplement: Supplementary file 1 — Additional file 1: Figure S1. RNA-seq analysis of CREB1 KO cells, CREB1 rescue cells, and WT cells. A Hierarchical clustering heatmap showing overall similarity between samples based on Euclidean distance calculation between CREB1 KO vs WT and Rescue vs KO. B Principal component analysis (PCA) showing sample variance based on gene expression. Points that represent samples were projected onto the 2D plane. Sample variance was indicated by Variance Percent. C, D Summary of DEGs in paired comparison of CREB1 KO vs WT and Rescue vs KO respectively (R upset package). Side horizontal bars indicate the total number of DEGs of each paired comparison. Vertical bars indicate the total number of DEGs exclusively existed in indicated combinations of paired comparisons. Figure S2. Rank-rank analysis of CREB1 rescue vs CREB1 KO gene ranks and tumor vs para-carcinoma tissue gene ranks. Rank–rank hypergeometric overlap (RRHO) plots of lists of 24 types of cancer vs 1, 2, 3 separately. Figure S3. Integrative analysis of CREB1-downregulated gene network. A Gene distribution analysis of four different gene lists (R circlize package). They are (i) CREB1 commonly downregulated gene list; (ii) RRHO concordant downregulated gene list; (iii) commonly opposite regulated genes between Rescue vs KO and KO vs WT; (iv) CREB1 Chipseq gene list (genes with p value ≤ 0.001, CREB1 binding ratio ≥ 2 are selected). The stripes of different colors connect the portions uniquely shared by different combinations of the lists (i–iv) (Additional file 5: Table S4). 1 gene (red stripe) are present in all 4 lists. 19 genes (blue stripe) are uniquely shared by i, ii, and iv. B. 20 top-ranked CREB1-downregulated targets illustrated by their average log2 FC (CREB1 rescue vs CREB1 KO) and the tumor occurrence number in RRHO concordant gene list. The scale of log2 FC is displayed by the tomato gradient bar. C, D. CREB1 binding sites in Chipseq data, gene function annotation based on GO analysis and detailed tumor [file 12935_2021_2224_MOESM1_ESM.pdf]
